# Effect of fasting-mimicking diet on markers of autophagy and metabolic health in human subjects

**DOI:** 10.1007/s11357-025-02035-4

**Published:** 2025-12-11

**Authors:** Sara E. Espinoza, Sue Park, Gavin Connolly, Wenbo Qi, Ning Zhang, Manpreet Semwal, Yan Li, Marie Lauzon, Adam B. Salmon, William Hsu, Min Wei, Nicolas Musi

**Affiliations:** 1 Center for Translational Geroscience, Department of Medicine, Cedars-Sinai Health Sciences Center, 8700 Beverly Boulevard, Los Angeles, CA 90048 USA; 2 Sam and Ann Barshop Institute for Longevity & Aging Studies, UT Health San Antonio, San Antonio, TX USA; 3 Biostatistics Shared Resource, Cedars-Sinai Health Sciences Center, Los Angeles, CA USA; 4 Departments of Molecular Medicine and Cellular and Integrative Physiology, UT Health San Antonio, San Antonio, TX USA; 5 Geriatric Research, Education, and Clinical Center, South Texas Veterans Healthcare System, San Antonio, TX USA; 6 L-Nutra, Inc., Culver City, CA USA

**Keywords:** Dietary intervention, Clinical trial, Autophagy, Fasting-mimicking diet

## Abstract

We conducted a pilot randomized clinical trial to examine the effects of the fasting-mimicking diet (FMD) on autophagic flux and metabolic health markers in healthy humans. Thirty healthy participants were randomized to two oral formulations of FMD (ProLon and FMD2) or control for 8 days. Blood was collected at baseline, days 4 and 6 during intervention, and 48 h after completing FMD (Day 8). Effects on autophagic flux were measured in peripheral blood mononuclear cells (PBMC) using the ratio of LC3B-II/LC3B-I protein in samples treated with chloroquine (CQ) *ex vivo.* Metabolic outcomes measured included fasting plasma glucose, insulin, insulin growth factor-1, β-hydroxy-butyrate (BHB), and insulin resistance (HOMA-IR). One-way ANOVA or Kruskal-Wallis tests were used to determine significant differences between groups, both at individual time points and changes from baseline to subsequent time points. Participants were 49.1 ± 11.8 years. Significant between-group differences were observed in changes from baseline to the end of the 6-day dietary intervention for body weight, fasting glucose, BHB, HOMA-IR, and autophagic flux (*p* < 0.05); however, differences were not significant across all time points. These results suggest that FMD may improve autophagic flux and markers of metabolic health. FMD may serve as a non-pharmaceutical intervention to modulate autophagy; however, further investigation with larger sample sizes is needed to confirm and extend these findings. This clinical trial was sponsored by L-Nutra Inc. and registered on ClinicalTrials.gov (NCT06115551).

**Graphical Abstract:**

**Figure:**
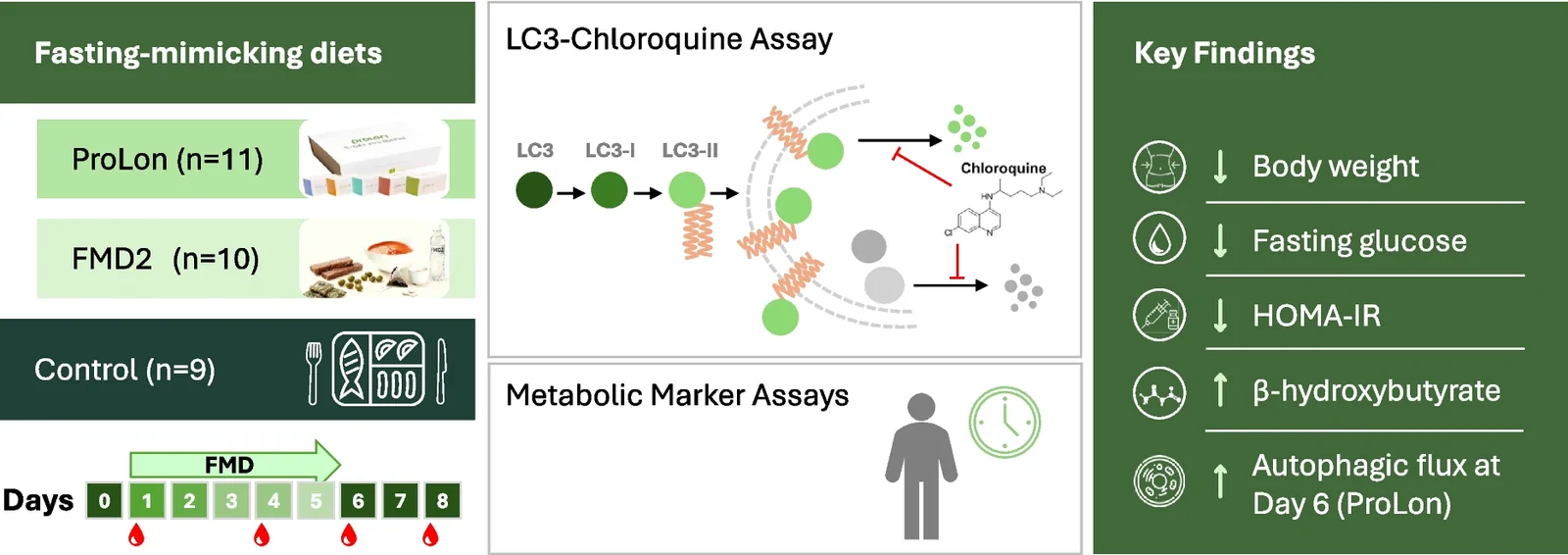

**Supplementary Information:**

The online version contains supplementary material available at https://doi.org/10.1007/s11357-025-02035-4.

## Introduction

Calorie restriction has numerous cardiometabolic health benefits in humans [1] and has been found to extend lifespan in multiple species [2]. However, adherence to a significantly calorie-restricted diet over the long term is difficult and carries potential risks [3]. Thus, other regimens of dietary modification and restriction have been investigated as potential options for similar health benefits [4, 5]. The fasting-mimicking diet (FMD) is a type of periodic, short-term dietary intervention that is intended to induce the endocrine and metabolic effects of water-only fasting while providing modest calories and essential nutrients [6–10]. FMD is a cyclic regimen consisting of two phases: (1) a 5-day dietary intervention that is low in overall calories, plant-based, low in protein, low in sugars, but high in unsaturated fats, and (2) a re-feeding period with a normal diet.

Randomized control trials in healthy adults have demonstrated that FMD leads to reduced total body fat and improved blood pressure, biological and immune system age, and biomarkers of diabetes and cardiovascular disease [6, 7, 11]. FMD has also been examined in patients with chronic diseases, such as multiple sclerosis, diabetes, Parkinson’s Disease, inflammatory bowel disease, breast cancer, and Alzheimer’s Disease, with suggestion of potential benefits [9, 12–16]. Therefore, FMD is considered a potential geroscience-based intervention to promote the extension of healthspan with aging [5]. The molecular targets of calorie restriction include multiple pathways leading to the hallmarks of aging, including promoting repair and recycling pathways, such as autophagy [5, 17]. The fasting and refeeding nature of FMD too is believed to affect these pathways [17]; however, few studies have examined whether FMD results in changes in autophagy in humans. Here, we report results of a randomized controlled trial of two FMD formulations compared to a control diet in healthy adults to examine FMD’s effects on both physiological and molecular outcomes by addressing metabolic changes and autophagic flux.

## Methods

### Study participants

Thirty participants were recruited from the San Antonio, Texas USA community. At an initial screening visit, medical history, physical exam, electrocardiogram, and clinical labs (complete blood count [CBC], comprehensive metabolic panel [CMP], lipid panel) were assessed to determine eligibility. Exclusion criteria were on the basis of medical history (gastric bypass, type 1 diabetes, other at-risk medical conditions assessed by the physician investigator), recent weight loss interventions (weight loss > 5%, weight loss medication, or weight loss program), therapies (immunosuppressant drugs, diabetes treatment other than diet or metformin), pregnancy, allergies to study foods, and alcohol dependency. All participants provided signed informed consent before participating in study procedures. The study was approved by the Institutional Review Board of the University of Texas Health Science Center at San Antonio.

### Study design

We conducted an open-label, randomized clinical trial with 1:1:1 parallel assignment to one of three arms: ProLon, FMD2, or control. After screening and consent, participants were randomized into one of the three groups via a block randomization scheme. Participants were instructed to consume only the foods of their assigned group, with no outside food or drinks (except water).

The total study participation was 8 days, with a total of four visits (visit 1: baseline, visit 2: Day 4, visit 3: Day 6, visit 4: Day 8). During each visit, vitals collection, waist/hip measurements, and blood draws were conducted. Blood was drawn for safety clinical labs (complete blood cell count, comprehensive metabolic profile), metabolic markers (fasting plasma glucose, insulin, insulin-like growth factor-1 [IGF-1], ketones [β hydroxy-butyrate]), and assessment of autophagic flux in peripheral blood mononuclear cells (PBMC). The lipid panel was collected only at baseline and Day 8. Metabolites, hormones, and lipids were measured by Quest Diagnostics (Secaucus, NJ). Insulin sensitivity was assessed with the homeostatic model assessment for insulin resistance (HOMA-IR) calculated as HOMA-IR = (fasting plasma insulin × fasting plasma glucose)/22.5.

### Intervention

Participants randomized to ProLon or FMD2 received 5-day meal replacement kits. The ProLon kit is the original FMD formulation evaluated in various trials [18, 19]. The FMD2 is a specialized low-starch formulation designed for better postprandial glucose control, with potential applications for individuals managing metabolic conditions and diabetes. Participants randomized to the control arm consumed their normal diet throughout the study period. All FMD diet kits were prepared by and provided by L-Nutra based on proprietary formulations. The kits consist of plant-based soups, energy bars, snacks, teas, and supplements. Caloric intake is 1100 kcal (40–50% calorie restriction) on day 1, and 700–800 kcal (~ 70–80% calorie restriction) on days 2 to 5.

### Autophagic flux assay

The ratio of LC3B-II/LC3B-I protein in chloroquine (CQ)-treated versus untreated samples is used as a measure of autophagic flux [20]. We used a previously published assay [21] with some modifications. Peripheral blood was collected from an antecubital vein between 9 and 9:30 AM after a 10 h overnight fast. Four 3-mL blood samples were collected in heparin-coated tubes, inverted, and transferred into four separate conical tubes. To assess autophagic flux, samples (two tubes for each patient/time) were treated with 30 mL of 15 mM CQ (Sigma, St. Louis, MO) and incubated at 37 °C for 1.5 h. Control samples were treated with an equal volume of PBS and treated similarly. Following incubation, 4 mL of cold DPBS were added to each tube and mixed by inversion. Then 4mL of Lymphoprep (STEMCELL Technologies Inc, Cambridge, MA) were added beneath the blood-DPBS mixture with a syringe. The tubes were then centrifuged for 30 min at 800 × g at 4 °C. The PBMC layer was aspirated and transferred into 10 mL conical centrifuge tubes. Replicate samples were pooled, and each tube was then diluted with 2× volume of cold DPBS, inverted, and pelleted by centrifugation at 600× g for 10 min at 4 °C. The supernatant layer was discarded, and the pellet was resuspended in 1 mL of cold red blood cell lysis buffer (eBioscience, Waltham, MA). The tubes were placed on ice for 2–3 min before centrifuging at 600× g for 5 min at 4 °C with the brake on. The PBMC pellet was then resuspended with 5 mL of cold DPBS and centrifuged at 600× g for 5 min at 4 °C with the brake on. The pellet was then resuspended in 1 mL of cold DPBS, transferred to a 1.5 mL microcentrifuge tube, and centrifuged at 2000× g for 10 min at 4 °C. The pellet was snap-frozen in dry ice and stored at −80 °C.

### Western blotting

For protein extraction, 100–200 µL cold RIPA buffer (Cat#89901 Pierce) with protease inhibitor cocktail (100x, Pierce #78442) were added to the PBMC pellet, vortexed, and sonicated. Samples were centrifuged at 14,000 rpm at 4 °C for 10 min, and the supernatant was transferred to 0.6 mL microcentrifuge tubes. Western blotting was done using LC3B primary antibody (Cell Signaling Technology, Danvers, MA) diluted in 1:500 TBS-T (3% BSA) and IRDye 800CW goat anti-rabbit IgG secondary antibody (Li-COR Biotechnology, Lincoln, NE) 1:15,000 diluted in TBS-T (3% BSA). Proteins were transferred to a 0.2 µm nitrocellulose membrane, scanned using the Li-COR Image System (Lincoln, NE), and analyzed with Image Studio software.

### Statistical analysis

Demographic, clinical, and autophagy assay measures were summarized at each time point using frequency and percentage for categorical variables and mean ± standard deviation for continuous variables. Depending on the normality of the data and test conditions, either a one-way ANOVA or Kruskal-Wallis test was run to determine significant between-group differences across the three intervention groups at each individual time point, as well as for changes from baseline to subsequent time points. All statistical analyses were conducted using SASv9.4. All tests were two-sided, and *p*-values less than 0.05 were considered statistically significant.

## Results

### Participant characteristics

Participants were enrolled across the three study arms: ProLon (*n* = 11), FMD2 (*n* = 10), and control (*n* = 9). Participants were aged 25–65 years, with a mean age of 49.1 ± 11.8 years; 83.3% were female and 83.3% Hispanic/Latino (see Table 1). Mean body mass index (BMI) was 28.6 ± 4.0 kg/m^2^, which significantly differed by intervention arm (*p* = 0.0039), being higher in the ProLon group (29.3 ± 4.1 kg/m^2^) and FMD2 (30.9 ± 2.1 kg/m^2^) groups compared to the control group (25.3 ± 3.6 kg/m^2^). There were no significant differences among the intervention groups at baseline for fasting glucose, insulin, IGF-1, BHB, or HOMA-IR. Furthermore, there were no significant differences in baseline measures of autophagy in PBMC among the treatment groups at baseline.

**Table 1:** Baseline characteristics

	ProLon *N* = 11	FMD2 *N* = 10	Control *N* = 9	Total *N* = 30	*P*-value
	*Mean (standard deviation) or n (%)*				
Age, years (range: 25.0–65.0)	52.3 (12.3)	49.2 (6.5)	45.0 (15.4)	49.1 (11.8)	0.23
Female, *n* (%)	8 (72.7)	9 (90.0)	8 (88.9)	25 (83.3)	0.58
Ethnoracial group					0.48
U.S. White, *n* (%)	1 (9.1)	3 (30.0)	1 (11.1)	5 (16.7)	
Hispanic/Latino, *n* (%)	10 (90.9)	7 (70.0)	8 (88.9)	25 (83.3)	
Weight, kg (range: 50.8–101.7)	79.0 (15.1)	80.4 (8.3)	66.3 (10.2)	75.6 (13.0)	**0.0278**
BMI, kg/m^2^ (range: 20.4–34.3)	29.3 (4.1)	30.9 (2.1)	25.3 (3.6)	28.6 (4.0)	**0.0039**
Fasting blood glucose, mg/dL (range: 73.0–112.0)	92.5 (4.1)	92.9 (11.0)	86.0 (8.6)	90.7 (8.6)	0.15
Insulin, mIU/L (range: 4.3–28.1)	11.8 (6.9)	12.0 (5.0)	8.9 (3.6)	11.0 (5.4)	0.37
IGF-1, ng/mL (range: 55.0–290.0)	119.5 (42.4)	111.3 (43.4)	161.6 (69.3)	129.4 (54.8)	0.10
Ketone, mmol/L (range: 0.0–0.2)	0.1 (0.0)	0.1 (0.1)	0.1 (0.0)	0.1 (0.0)	0.40
HOMA-IR (range: 0.9–7.1)	2.9 (1.8)	2.9 (1.5)	1.9 (0.7)	2.6 (1.5)	0.32
Untreated LC3BII/LC3BI (range: 0.4–2.1)	1.1 (0.6)	0.8 (0.3)	0.7 (0.3)	0.9 (0.4)	0.18
CQ-treated LC3BII/LC3BI (range: 0.2–5.0)	2.0 (1.5)	2.3 (0.9)	2.0 (1.4)	2.1 (1.2)	0.88
CQ-treated/untreated LC3BII/LC3BI ratio (range: 0.2–6.4)	2.2 (1.8)	3.0 (1.6)	3.3 (2.1)	2.8 (1.8)	0.42

### Metabolic health markers

**Fig. 1:**
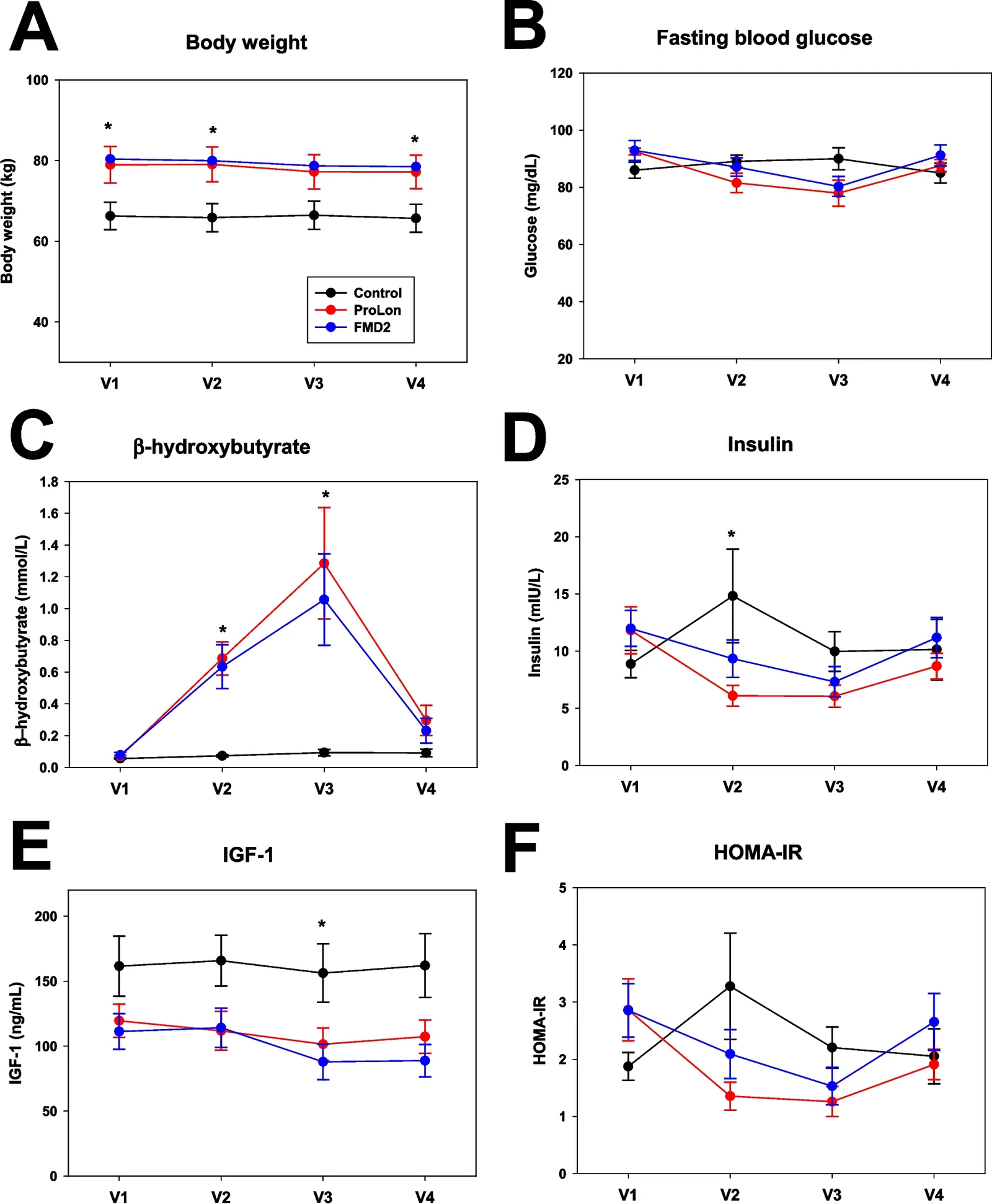
The effect of FMD intervention (ProLon, FMD2) and control on metabolic parameters, including body weight, fasting blood glucose, β-hydroxybutyrate, insulin, IGF-1, and HOMA-IR. * Denotes statistically significant differences observed among groups at the indicated timepoint

Significant differences from baseline to the end of the dietary intervention at 6 days were observed between treatment groups, with an increase in β-hydroxybutyrate and decreases in weight, fasting glucose, insulin, and HOMA-IR in the FMD groups compared to the control group (Table 2). However, no significant differences in fasting glucose or HOMA-IR were noted between the groups at individual visits (Supplemental Table 1). The HOMA-IR was found to increase in the control group from visit 1 to visit 2, while both FMD groups showed a decrease in HOMA-IR (Supplemental Table 2). Trends in metabolic health markers are shown in Fig. 1.

**Table 2:** Change from baseline to end of fasting-mimicking diet at 6 days

	ProLon *N* = 11	FMD2 *N* = 10	Control *N* = 9	***P*-value**
	*Mean change from baseline (standard deviation)*			
Weight, kg	−1.7 (1.6)	−1.7 (1.0)	0.2 (0.8)	**0.0003**
Fasting glucose, mg/dL	−14.4 (12.7)	−12.6 (10.8)	4 (3.2)	**0.0003**
Ketone (β-hydroxybutyrate), mmol/L	1.2 (1.2)	1.0 (0.9)	0.0 (0.1)	**0.0005**
Insulin, mIU/L	−6.3 (5.8)	−4.7 (2.6)	1.1 (3.0)	**0.0016**
IGF-1, ng/mL	−23.5 (29.5)	−23.4 (21.1)	−5.3 (33.5)	0.2975
HOMA-IR	−1.6 (1.5)	−1.3 (0.8)	0.3 (0.7)	**0.0009**
Untreated LC3B-II/LC3B-I	−0.3 (0.4)	−0.2 (0.3)	0.5 (0.7)	**0.0035**
CQ-treated LC3B-II/LC3B-I	0.1 (1.8)	−0.8 (0.8)	0.6 (2.9)	0.3231
CQ-treated/untreated LC3B-II/LC3B-I ratio	1.9 (2.9)	−0.1 (2.0)	−1.1 (2.0)	0.0876

### Autophagic flux

We used PBMC to perform *ex vivo* assays of autophagic flux in patient samples. Macroautophagy is an autophagosome-mediated process that facilitates lysosomal degradation of intracellular components. Autophagy can be measured by the accumulation of LC3B-II, a lipidated LC3B-I that binds to both the inner and outer autophagosomal membranes. The amount of LC3B-II is generally proportional to the number of autophagosomes; thus, an increase in the LC3B-II/LC3B-I ratio may be indicative of autophagy activation. However, the inner-membrane bound LC3B-II is degraded by lysosomal proteases upon autophagosome fusion with the lysosome. A decrease in the LC3B-II/LC3B-I ratio suggests enhanced autophagic degradation.

**Fig. 2:**
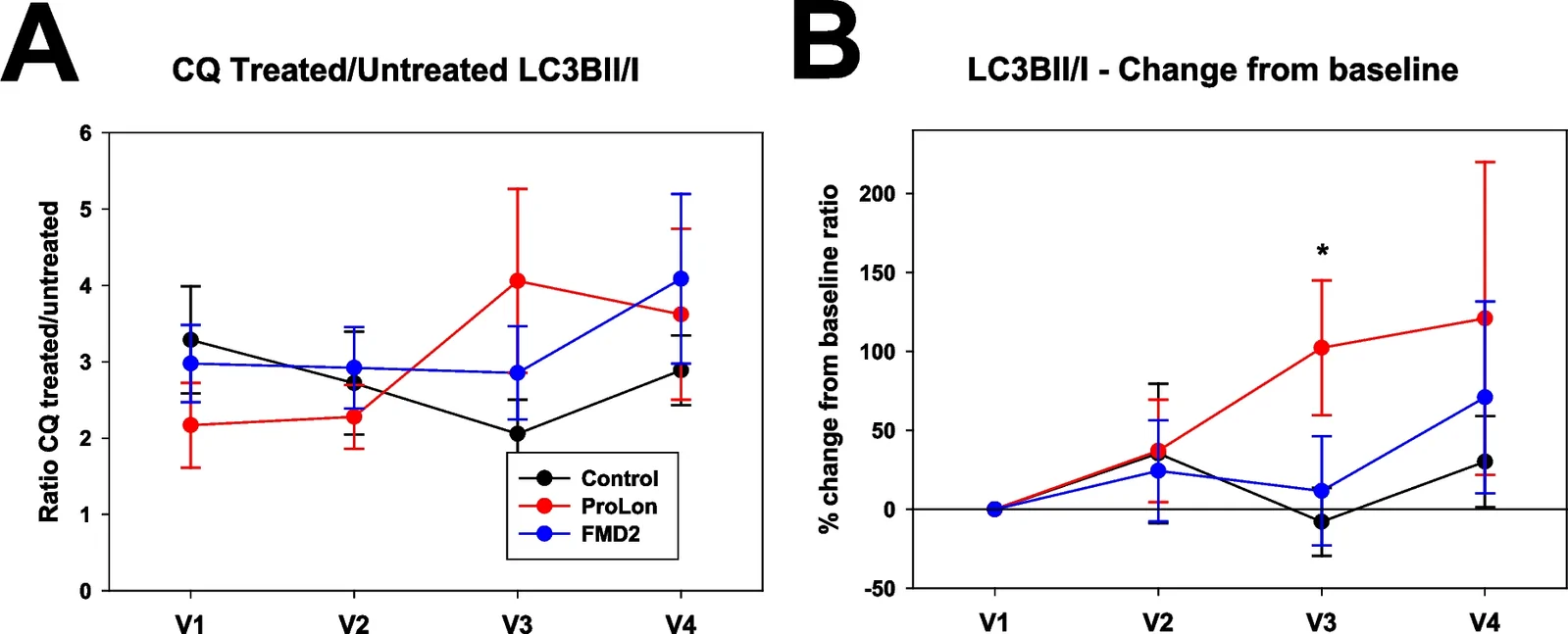
The effect of FMD intervention (ProLon, FMD2) and control on measures of autophagic flux

An increase in the LC3B-II/LC3B-I ratio following CQ treatment points to enhanced autophagic flux. In the absence of CQ, a higher LC3B-II/LC3B-I ratio reflects increased autophagic induction, whereas a lower ratio suggests elevated autophagic degradation. Trends in the CQ-treated and untreated LC3B-II/LC3B-I ratios across study visits are illustrated in Fig. 2. No differences were observed for the CQ-untreated LC3B-II/LC3B-I ratio, a direct marker of autophagosome accumulation, among the three groups (*p* = 0.1810; Supplemental Table 1). By Day 6, the control group exhibited a significantly higher mean ratio (1.3 ± 0.7) compared to both ProLon (0.7 ± 0.4) and FMD2 (0.7 ± 0.4) groups (*p* = 0.0191), and this trend persisted through Day 8 with a near-significant difference (*p* = 0.0556). These findings suggest that FMD enhances autophagic flux through increased autophagic degradation (Fig. 2), consistent with the observation that the CQ-treated LC3B-II/LC3B-I ratio (i.e., LC3-II levels under lysosomal inhibition) did not differ among the three groups over the course of intervention (Table S1**)**. To measure autophagic flux, the rate of autophagic degradation rather than the number of autophagosomes, we used the ratio of LC3B-II/LC3B-I in CQ-treated versus CQ-untreated samples. Trends in autophagy flux at various study visits are illustrated in Fig. 2B. A trend toward increased autophagic flux was observed on Day 6, and this elevated level persisted after 2 days of normal feeding on Day 8 in the ProLon group. For the FMD2 group, the elevated trend in autophagy flux was observed only 2 days post intervention.

## Discussion

In this randomized control trial of two formulations of FMD, we observed improvements in metabolic health markers in both ProLon and FMD2 groups compared to the control group. In the ProLon group only, we observed an increase in autophagic flux as measured by a decrease in untreated LC3B-II/LC3B-I from baseline at Day 6 with *ex vivo* modulation of PBMC following the FMD intervention. More studies are needed to explore the mechanisms underlying FMD-induced cellular responses and to evaluate the long-term effects of various FMD formulations in the intended metabolic or diabetes patients. Our findings are in line with prior clinical studies, which have shown that FMD has beneficial effects on various metabolic health markers such as fasting glucose, HOMA-IR, and ketones [22], and preclinical studies of FMD that have shown that short-term fasting induces autophagy in rodents [10, 23]. Therefore, there is interest in FMD as an intervention for the treatment of age-related diseases and to extend healthspan [8, 9, 11–16, 24, 25].

Caloric restriction has long been known to extend lifespan in several preclinical models [26] and to have health benefits in human subjects [2, 27]. Due to the challenges and potential detrimental effects in maintaining calorie restriction over time, there is considerable interest in alternative methods for dietary restriction, including periodic fasting. FMD, a type of periodic fasting, has been investigated as a form of dietary restriction that may be more feasible in terms of adherence, as it includes modest short-term dietary restriction.

FMD has shown potential benefits for chronic diseases and conditions, such as metabolic syndrome, fatty liver disease, multiple sclerosis, diabetes, Parkinson’s disease, cancer, and Alzheimer’s disease in preclinical and emerging clinical studies [9, 12–16, 28–31]. Rodent studies have shown that FMD complements conventional cancer treatments by enhancing treatment efficacy and reducing tumor size [32–34] and inducing autophagy in cancer cells, making them more susceptible to chemotherapies [35]. FMD has also been shown to reduce amyloid β protein levels, indicative of decreased neuroinflammation, in rodent models of Alzheimer’s disease [28, 36]. Clinical studies show improvements in cognitive function and overall well-being in patients with Alzheimer’s after consuming three cycles of FMD [28]. Therefore, FMD may serve as a short-term dietary regimen providing potential health benefits without the adverse side effects from prolonged calorie restriction [8].

**Fig. 3:**
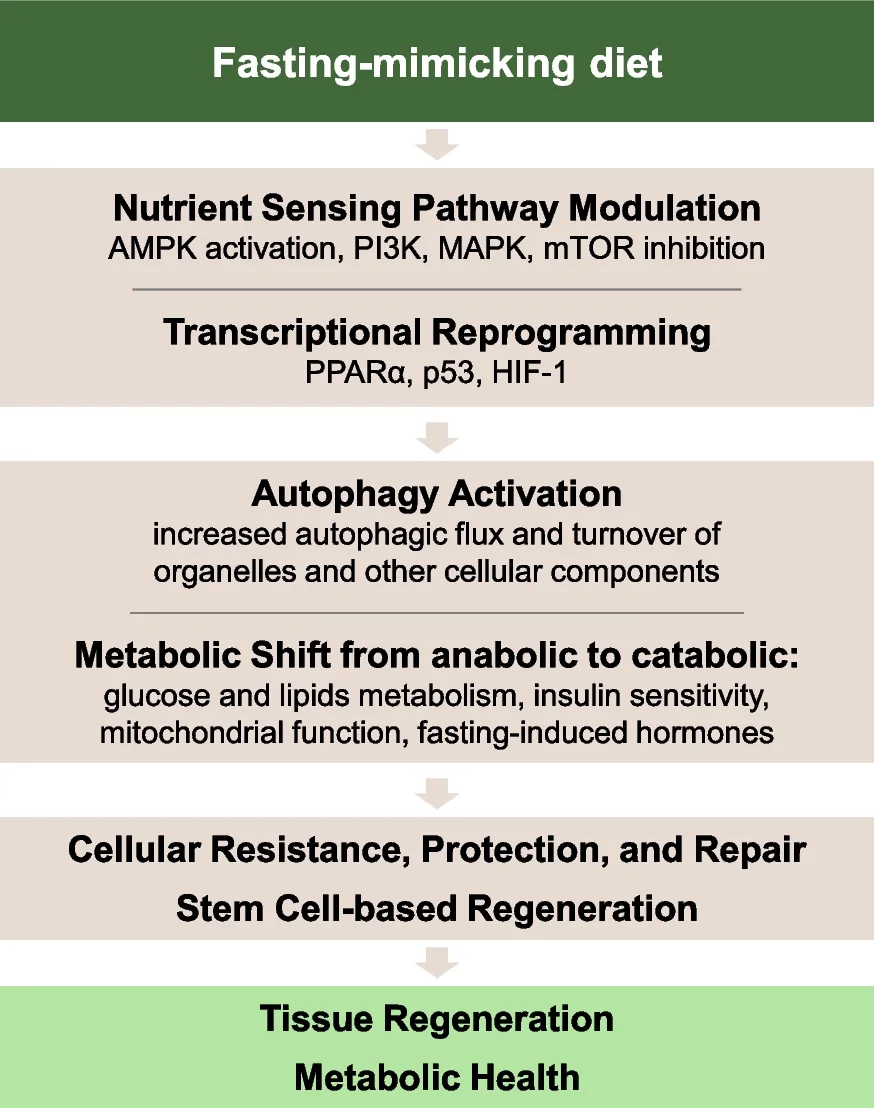
A schematic of hypothesized mechanism of effects of FMD on autophagy and metabolic health. * Denotes statistically significant differences observed among groups at the indicated timepoint

The hypothesized pathway from FMD to autophagy activation involves modulation of nutrient sensing pathways (i.e., AMPK activation, mTOR inhibition) and transcriptional reprogramming, which lead to a metabolic shift from anabolic to catabolic and increased autophagic flux and turnover of organelles and other cellular components (Fig. 3) [5, 17, 29, 37]. Similar to our findings, other randomized control trials of FMD in healthy adults have shown various health benefits, including reduced total body fat, blood pressure, fasting plasma glucose, circulating triglycerides, biological and immune system age, IGF-1, and improved biomarkers of diabetes and cardiovascular disease [6, 7, 11, 38, 39]. A recent clinical trial found that FMD, including either high or low protein content, led to improved metabolic health markers and induced autophagy as measured by the expression of genes involved in autophagy (*GABARAPL1*,* ATG2A*, *MAP1 LC3A*,* ZFPM1*) compared to the control diet [38]. However, some autophagy-related genes (*ULK1*,* BNIP3*) varied according to low versus high protein FMD. Interestingly, high-only protein FMD led to selectively reduced visceral fat mass, improved heart rate variability, and improved gut microbiome. These results suggest that the effects of FMD on overall physiology may vary by specific FMD nutrient composition, which could potentially be tailored based on needs and intended effects. However, larger studies will be needed to further examine these possibilities.

Autophagy, a lysosomal-mediated degradation process, is essential for cellular homeostasis by maintaining innate and adaptive immunity, mitochondrial function, intercellular communication, stem cell regeneration, cell senescence, telomeres, and stress resilience [40–42] and is a hallmark of aging and age-related disease [43]. Several studies suggest that autophagy plays a significant role in the longevity benefits observed with calorie restriction [44]. To the best of our knowledge, this is the first clinical trial examining the effect of FMD on autophagic flux measured by the ratio of LC3B-II/LC3B-I protein in PBMCs at several timepoints during and following dietary restriction. Autophagy is induced by stress through mTOR inhibition and AMPK activation [45]. At the cellular level, nutrient deprivation attenuates mTOR signaling, thereby increasing autophagic activity [46, 47]. It also enhances antioxidant pathways, induces apoptosis, downregulates inflammation [40, 48], and leads to improvements in acetylcholine-induced vasorelaxation [49]. Autophagy dysfunction has been implicated in neurodegenerative diseases, cancers, autoimmune disorders, and inflammatory conditions [50, 51]. Therefore, modulating autophagy is a potential therapeutic target for targeting aging itself as well as a host of age-related diseases.

Limitations of this study include its small sample size and the short assessment period of one cycle of FMD. Therefore, we were unable to assess more long-term effects of repeated FMD cycles on autophagic and metabolic markers or other markers of health outcomes. We also note that, by chance, the body weight and BMI were higher in the ProLon and FMD2 groups compared to the control groups at baseline prior to the intervention, which may have impacted our findings. Preclinical studies have found that baseline body weight and adiposity may influence the longevity effects of dietary restriction [27]; however, we did not find an association between body weight and autophagic flux in our study. Future studies with longer intervention and follow-up periods as well as targeted populations (e.g., FMD 2 for diabetic patients) may offer insight into the duration of FMD-induced autophagic and metabolic changes.

In summary, this pilot study of FMD in healthy adults found that FMD leads to improvements in metabolic health parameters and that one FMD formulation, ProLon, may improve autophagic flux. Our results suggest that calorie restriction such as that used in the FMD may be of potential benefit to modulate aging mechanisms such as autophagy to reduce the onset of age-related diseases and extend healthspan.

## Supplementary Information

Below is the link to the electronic supplementary material.

ESM 1 (DOCX 22.5 KB)

## Data Availability

The data underlying this study may be made available to qualified investigators upon reasonable request and review by the corresponding author.
