# Supplementary material for: Effect of fasting-mimicking diet on markers of autophagy and metabolic health in human subjects
Source: GeroScience. 2025 Dec 11;48(5):7083–93. doi: 10.1007/s11357-025-02035-4 (PMC13601415; doi:10.1007/s11357-025-02035-4)
Supplement: Supplementary file 1 [file 11357_2025_2035_MOESM1_ESM.docx]

| **Supplemental Table 1. All variables at all time points by intervention group** | | | | |
| --- | --- | --- | --- | --- |
|  | **ProLon**  N =11 | **FMD2**  N =10 | **Control**  N =9 | **P-value** |
|  | *Mean (standard deviation)* | | |  |
| Weight, kg | | | | |
| Visit 1: Day 0 | 79.0 (15.1) | 80.4 (8.3) | 66.3 (10.2) | **0.0278** |
| Visit 2: Day 4 | 79.1 (14.2) | 80.0 (8.2) | 65.9 (10.5) | **0.0206** |
| Visit 3: Day 6 | 77.2 (14.1) | 78.7 (8.2) | 66.5 (10.5) | 0.0527 |
| Visit 4: Day 8 | 77.2 (13.8) | 78.5 (8.0) | 65.7 (10.3) | **0.0362** |
| Fasting glucose, mg/dL | | | | |
| Visit 1: Day 0 | 92.5 (4.1) | 92.9 (11.0) | 86.0 (8.6) | 0.1489 |
| Visit 2: Day 4 | 81.5 (11.3) | 87.1 (10.1) | 89.1 (6.5) | 0.2065 |
| Visit 3: Day 6 | 78.0 (15.1) | 80.3 (11.0) | 90.0 (11.5) | 0.1157 |
| Visit 4: Day 8 | 87.6 (7.2) | 91.2 (11.7) | 85.0 (10.4) | 0.4027 |
| Ketone (β-hydroxybutyrate) | | | | |
| Visit 1: Day 0 | 0.1 (0.0) | 0.1 (0.1) | 0.1 (0.0) | 0.4009 |
| Visit 2: Day 4 | 0.7 (0.3) | 0.6 (0.4) | 0.1 (0.0) | **0.0005** |
| Visit 3: Day 6 | 1.3 (1.2) | 1.1 (0.9) | 0.1 (0.1) | **0.0006** |
| Visit 4: Day 8 | 0.3 (0.3) | 0.2 (0.2) | 0.1 (0.1) | 0.0517 |
| Insulin, mIU/L | | | | |
| Visit 1: Day 0 | 11.8 (6.9) | 12.0 (5.0) | 8.9 (3.6) | 0.3660 |
| Visit 2: Day 4 | 6.1 (3.0) | 9.3 (5.2) | 14.8 (12.3) | **0.0383** |
| Visit 3: Day 6 | 6.1 (3.2) | 7.3 (4.2) | 10.0 (5.2) | 0.1451 |
| Visit 4: Day 8 | 8.7 (3.7) | 11.2 (5.6) | 10.1 (7.9) | 0.6008 |
| IGF-1 | | | | |
| Visit 1: Day 0 | 119.5 (42.4) | 111.3 (43.4) | 161.6 (69.3) | 0.1000 |
| Visit 2: Day 4 | 111.8 (49.5) | 114.2 (48.1) | 165.8 (58.7) | 0.0695 |
| Visit 3: Day 6 | 101.5 (41.5) | 87.9 (43.1) | 156.2 (67.5) | **0.0191** |
| Visit 4: Day 8 | 107.3 (42.3) | 88.8 (39.8) | 162.0 (73.8) | 0.0501 |
| HOMA-IR | | | | |
| Visit 1: Day 0 | 2.9 (1.8) | 2.9 (1.5) | 1.9 (0.7) | 0.3201 |
| Visit 2: Day 4 | 1.4 (0.8) | 2.1 (1.3) | 3.3 (2.8) | 0.0526 |
| Visit 3: Day 6 | 1.3 (0.9) | 1.5 (1.0) | 2.2 (1.1) | 0.1266 |
| Visit 4: Day 8 | 1.9 (0.9) | 2.7 (1.6) | 2.1 (1.4) | 0.6986 |
| Untreated LC3B-II/LC3B-I | | | | |
| Visit 1: Day 0 | 1.1 (0.6) | 0.8 (0.3) | 0.7 (0.3) | 0.1810 |
| Visit 2: Day 4 | 0.9 (0.4) | 0.7 (0.4) | 1.1 (0.7) | 0.2287 |
| Visit 3: Day 6 | 0.7 (0.4) | 0.7 (0.3) | 1.3 (0.7) | **0.0191** |
| Visit 4: Day 8 | 1.0 (0.5) | 0.5 (0.3) | 1.0 (0.5) | 0.0556 |
| CQ-Treated LC3B-II/LC3B-I | | | | |
| Visit 1: Day 0 | 2.0 (1.5) | 2.3 (0.9) | 2.0 (1.4) | 0.8812 |
| Visit 2: Day 4 | 1.7 (1.1) | 1.6 (0.3) | 2.2 (1.2) | 0.3349 |
| Visit 3: Day 6 | 2.1 (1.6) | 1.5 (0.5) | 2.5 (2.4) | 0.6951 |
| Visit 4: Day 8 | 2.4 (2.0) | 1.4 (0.4) | 2.7 (1.6) | **0.0436** |
| CQ-Treated/Untreated LC3B-II/CL3B-I | | | | |
| Visit 1: Day 0 | 2.2 (1.8) | 3.0 (1.6) | 3.3 (2.1) | 0.4227 |
| Visit 2: Day 4 | 2.3 (1.4) | 2.9 (1.7) | 2.7 (2.0) | 0.7050 |
| Visit 3: Day 6 | 4.1 (4.0) | 2.9 (1.9) | 2.1 (1.4) | 0.5588 |
| Visit 4: Day 8 | 3.6 (3.7) | 4.1 (3.5) | 2.9 (1.4) | 0.7345 |

| **Supplemental Table 2. Change in all variables at all follow-up time points compared to baseline** | | | | |
| --- | --- | --- | --- | --- |
|  | **ProLon** | **FMD2** | **Control** | **P-value** |
|  | *Mean (standard deviation)* | | |  |
| Weight, kg | | | | |
| Visit 2: Day 4 | 0.1 (1.6) | -0.4 (1.2) | -0.4 (0.9) | 0.1046 |
| Visit 3: Day 6 | -1.7 (1.6) | -1.7 (1.0) | 0.2 (0.8) | **0.0003** |
| Visit 4: Day 8 | -1.8 (1.6) | -1.9 (1.0) | -0.6 (0.9) | **0.0191** |
| Fasting glucose, mg/dL | | | | |
| Visit 2: Day 4 | -11.0 (9.6) | -5.8 (9.0) | 3.1 (4.7) | **0.0029** |
| Visit 3: Day 6 | -14.4 (12.7) | -12.6 (10.8) | 4.0 (3.2) | **0.0003** |
| Visit 4: Day 8 | -4.8 (5.6) | -1.7 (5.8) | -1.0 (6.7) | 0.3480 |
| Ketone (β-hydroxybutyrate), mmol/L | | | | |
| Visit 2: Day 4 | 0.6 (0.3) | 0.6 (0.4) | 0.0 (0.0) | **0.0005** |
| Visit 3: Day 6 | 1.2 (1.2) | 1.0 (0.9) | 0.0 (0.1) | **0.0005** |
| Visit 4: Day 8 | 0.2 (0.3) | 0.2 (0.2) | 0.0 (0.1) | 0.0760 |
| Insulin, mIU/L | | | | |
| Visit 2: Day 4 | -5.7 (5.9) | -2.7 (4.6) | 6.0 (12.6) | **0.0156** |
| Visit 3: Day 6 | -6.3 (5.8) | -4.7 (2.6) | 1.1 (3.0) | **0.0016** |
| Visit 4: Day 8 | -3.6 (5.3) | -0.8 (1.9) | 1.3 (5.6) | 0.1530 |
| IGF-1, ng/mL | | | | |
| Visit 2: Day 4 | -7.7 (36.1) | 2.9 (16.6) | 4.2 (19.9) | 0.5331 |
| Visit 3: Day 6 | -23.5 (29.5) | -23.4 (21.1) | -5.3 (33.5) | 0.2975 |
| Visit 4: Day 8 | -17.7 (30.0) | -22.5 (18.1) | 0.4 (19.2) | 0.0984 |
| HOMA-IR | | | | |
| Visit 2: Day 4 | -1.5 (1.6) | -0.8 (1.3) | 1.4 (2.9) | **0.0098** |
| Visit 3: Day 6 | -1.6 (1.5) | -1.3 (0.8) | 0.3 (0.7) | **0.0009** |
| Visit 4: Day 8 | -1.0 (1.4) | -0.2 (0.4) | 0.2 (1.1) | 0.0973 |
| Untreated LC3B-II/LC3B-I | | | | |
| Visit 2: Day 4 | -0.2 (0.7) | -0.1 (0.3) | 0.3 (0.5) | 0.2646 |
| Visit 3: Day 6 | -0.3 (0.4) | -0.2 (0.3) | 0.5 (0.7) | **0.0035** |
| Visit 4: Day 8 | 0.1 (0.5) | -0.3 (0.3) | 0.2 (0.5) | **0.0449** |
| CQ-Treated LC3B-II/LC3B-I | | | | |
| Visit 2: Day 4 | -0.3 (1.3) | -0.7 (1.0) | 0.4 (0.9) | 0.1759 |
| Visit 3: Day 6 | 0.1 (1.8) | -0.8 (0.8) | 0.6 (2.9) | 0.3231 |
| Visit 4: Day 8 | 0.7 (2.1) | -0.9 (0.9) | 0.7 (2.1) | 0.1133 |
| CQ-Treated/Untreated LC3B-II/LC3B-I Ratio | | | | |
| Visit 2: Day 4 | 0.1 (1.8) | -0.1 (2.4) | -0.3 (2.0) | 0.9225 |
| Visit 3: Day 6 | 1.9 (2.9) | -0.1 (2.0) | -1.1 (2.0) | 0.0876 |
| Visit 4: Day 8 | 1.4 (3.4) | 1.4 (3.5) | -0.2 (1.8) | 0.5637 |
